# Diagnostic assistance provided by a pharmacist for the syndrome of inappropriate antidiuretic hormone secretion caused by carboplatin plus nab-paclitaxel chemotherapy in an elderly patient with lung cancer: a case report

**DOI:** 10.1186/s40780-025-00441-6

**Published:** 2025-04-23

**Authors:** Hayahide Ooi, Yuki Asai, Yasumasa Sakakura, Masaaki Takahashi

**Affiliations:** 1 Pharmacy, National Hospital Organization Mie Chuo Medical Center, 2158-5 Hisaimyojincho, Tsu, Mie 514-1101 Japan; 2 Department of Pharmacy, Mie University Hospital, Faculty of Medicine, Mie University, 2-174 Edobashi, Tsu, Mie 514-8507 Japan; 3 Department of pulmonary Medicine, National Hospital Organization Mie Chuo Medical Center, 2158-5 Hisaimyojincho, Tsu, Mie 514-1101 Japan

**Keywords:** Lung cancer, Syndrome of inappropriate antidiuretic hormone secretion, Carboplatin, Nab-paclitaxel

## Abstract

****Background**:**

Syndrome of inappropriate antidiuretic hormone secretion (SIADH) is the most common cause of hyponatremia. Although SIADH induced by carboplatin (CBDCA) plus nab-paclitaxel (nab-PTX) has been reported, there is limited evidence for SIADH being suspected by pharmacists during chemotherapy in elderly patients and contributing to early intervention through diagnostic support for physicians.

**Case presentation:**

An 84-year-old man was diagnosed with stage 3A squamous cell carcinoma of the right lung. Genetic mutations and expression of programmed cell death protein ligand 1 were < 1%. The patient was started on CBDCA area under the curve of 5 mg/mL·min on day 1 plus nab-PTX 70 mg/m^2^ on days 1, 8 and 15 once every 3 weeks. The serum sodium level immediately before the start of chemotherapy was 141 mmol/L. On day 8, it decreased to 119 mmol/L, and the physician started oral sodium chloride (3 g/day) administration. Because the pharmacist suspected that this hyponatremia may be due to chemotherapy-induced SIADH, the pharmacist suggested an examination of plasma and urine osmolality and urinary sodium levels to the physician. The serum creatinine level, plasma osmolality, urine osmolality, and urinary sodium level were 1.06 mg/dL, 253 mOsm/kg, 355 mOsm/kg, and 59 mEq/L, respectively; furthermore, the patient was not dehydrated. Based on the findings, a diagnosis of chemotherapy-induced SIADH was made. The physician and pharmacist conferred and decided to continue chemotherapy with frequent monitoring of serum sodium levels. Subsequently, the serum sodium level improved to 139 mmol/L on day 20 without additional treatment, and oral administration of sodium chloride was discontinued on day 22. The patient completed five cycles of chemotherapy. Computed tomography revealed a partial response throughout chemotherapy. Furthermore, sodium levels did not decrease again throughout chemotherapy. The Naranjo Adverse Drug Reaction Probability Scale score was 5 points, which is categorized as “probable.”

****Conclusions**:**

We encountered a case in which the patient developed chemotherapy-induced SIADH but was able to continue chemotherapy because of early pharmacist intervention. In elderly patients without genetic mutations and few treatment options, even if they develop SIADH, chemotherapy should be continued with monitoring of serum sodium levels by physicians and pharmacists.

## Background

Syndrome of inappropriate antidiuretic hormone secretion (SIADH) is the most common cause of hyponatremia [1]. It is characterized by euvolemic hyponatremia due to inappropriate retention of free water under the influence of antidiuretic hormones and manifests as symptoms such as water retention, increased urinary excretion, and dilutional hyponatremia [2]. Hyponatremia is associated with increased mortality and longer hospital stay in hospitalized patients [3]. Mild SIADH is characterized by symptoms such as nausea, vomiting, weakness, headache, and mild neurocognitive deficits, while severe disease often causes delirium, confusion, impaired consciousness, ataxia, and seizures, which could result in death [4]. Although solid tumors, pulmonary illnesses, and central nervous system disorders are known to cause SIADH, various drugs may also contribute to its development [5]. A retrospective study revealed that among patients with SIADH, 10% (44/439) were reported to have SIADH caused by drugs, and 6.8% (3/44) of cases of drug-induced SIADH were attributable to anticancer drugs [6]. In an analysis of 29 phase II and III trials, the incidence of severe hyponatremia (sodium level: < 130 mmol/L) among patients receiving platinum-based chemotherapy was reported to be 11.9% (266/2238) [7]. In particular, a multicenter randomized phase III study revealed that the incidence of grade 3 or 4 hyponatremia, classed according to the Common Terminology Criteria for Adverse Events, associated with carboplatin (CBDCA) plus nab-paclitaxel (nab-PTX), was 0.4% (2/521) [8].

Because chemotherapy tolerance generally decreases in elderly patients, the balance between efficacy and tolerability is important [9]. A multicenter phase III randomized trial indicated that despite increased adverse drug reactions such as decreased neutrophil count, platinum-based doublet chemotherapy was associated with survival benefits compared with vinorelbine or gemcitabine monotherapy in elderly patients with non-small cell lung cancer [10]. Whereas some cases of SIADH induced by CBDCA and PTX have been reported [11, 12], the affected patients were relatively young (age: ≤ 65 years).

Although SIADH is likely to lead to serious clinical outcomes in elderly patients, cognitive impairment may delay the timely identification of symptoms [13]. Therefore, proactive assessments of patients’ subjective symptoms and monitoring of blood test results by healthcare workers are crucial. Early pharmacist interventions have been reported to be useful for the detection of adverse drug reactions and exacerbation avoidance, including electrolyte abnormalities [14]. However, the diagnosis of SIADH-associated hyponatremia is challenging because of the nonspecific nature of its symptoms, the wide range of potential causes of hyponatremia, and the necessity to use appropriate diagnostic criteria for accurate evaluation. Without a thorough assessment using proper diagnostic tools, it can be difficult to definitively diagnose SIADH. To the best of our knowledge, there are limited case reports of pharmacists suspecting SIADH during chemotherapy in elderly patients and contributing to early intervention through diagnostic support for physicians.

In this case report, we describe the case of an elderly man who was able to complete CBDCA plus nab-PTX therapy without regimen change after the pharmacist assisted the diagnosis of chemotherapy-induced SIADH.

## Case presentation

The patient was an 84-year-old man (height: 168.6 cm, body weight: 61.5 kg, body mass index: 21.6 kg/m^2^, body surface area: 1.70 m^2^) who was diagnosed with stage 3A squamous cell carcinoma of the right lung 1 month ago. He tested negative for *EGFR* 19del, *EGFR* L858R, *BRAF* V600E, *KRAS* G12C, *ALK* fusion, *ROS1* fusion, *MET* exon 14 skipping, and *RET* fusion, and the expression of programmed cell death protein ligand 1 was < 1%. His medical history included hyperuricemia, gastric ulcer, atrial fibrillation, hypertension, and inguinal hernia. He had been taking the following medications: allopurinol 200 mg/day, famotidine 20 mg/day, edoxaban 60 mg/day, mefruside 25 mg/day, limaprost alfadex 15 μg/day, and triazolam 0.5 mg/day. The patient had no history of allergies or adverse drug reactions. His baseline clinical laboratory data are shown in Table 1. The attending physician referred the patient to the radiology department; however, definitive radiation therapy was deemed inappropriate because of suspected pleural dissemination and lymphangitis carcinomatosis. Therefore, the patient received chemotherapy alone.

**Table 1:** Clinical laboratory data immediately before each cycle of chemotherapy and the day of onset SIADH

Factors	day 0 (Cycle 1)	day 8 (Cycle 1)	day 44 (Cycle 2)	day 79 (Cycle 3)	day 111 (Cycle 4)	day 147 (Cycle 5)
Albumin (g/dL)	3.6	-	3.9	3.4	3.8	3.5
ALT (U/L)	14	20	14	12	13	13
AST (U/L)	24	34	23	18	18	21
ALP IFCC (U/L)	76	82	81	82	102	96
γ-GTP (U/L)	24	30	29	28	34	31
LDH IFCC (U/L)	201	180	220	183	231	224
T-bilirubin (mg/dL)	1.0	1.9	1.1	0.8	0.7	0.7
Na (mmol/L)	141	119	140	141	141	142
Cl (mmol/L)	105	83	105	108	104	108
K (mmol/L)	3.7	4.0	3.9	4.3	4.0	4.6
BUN (mg/dL)	25.9	33.8	28.0	25.7	21.0	18.6
Creatinine (mg/dL)	1.16	1.06	1.26	1.09	1.17	0.97
eGFR (mL/min/1.73 m^2^)	46.2	50.9	42.1	49.3	45.7	56.0
C-reactive protein (mg/dL)	1.06	4.00	0.16	0.26	3.04	0.50
White blood cell (× 10^2^/µL)	54.2	42.5	56.3	29.6	43.4	29.2
Red blood cell (× 10^4^/µL)	431	422	413	323	355	310
Hemoglobin (g/dL)	14.3	13.7	13.8	10.7	11.6	10.3
Hematocrit (%)	43.1	39.5	42.1	33.2	36.8	32.6
Platelet (× 10^4^/μL)	18.5	15.4	17.2	14.6	16.1	13.4
Neutrophil (× 10^2^/µL)	38.7	34.1	35.1	18.4	25.4	14.7
Monocytes(× 10^2^/µL)	2.7	0.7	2.8	1.7	2.6	1.4

*Abbreviations*: *ALP* Alkaline phosphatase, *ALT* Alanine aminotransferase, *AST* Aspartate aminotransferase, *BUN* Blood urea nitrogen, *Cl* Chloride, *GFR* Estimate glomerular filtration rate, *K* Potassium, *LDH* Lactate dehydrogenase, *Na* Sodium, *γ-GTP* Gamma-glutamyl transpeptidase

**Fig. 1:**
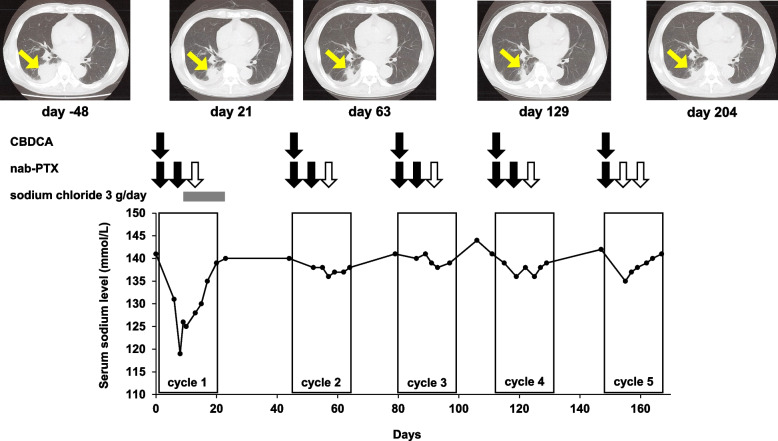
Summary of serum sodium levels, chemotherapy course, and computed tomography images. The x-axis indicates the number of days and day 0 indicates the first day of hospitalization. The y-axis indicates the serum sodium levels. Black and white arrows indicate the days of chemotherapy administration and discontinuation owing to neutropenia, respectively. The yellow arrows indicate the tumors. CBDCA, carboplatin; PTX, paclitaxel

A month after the diagnosis, first-line chemotherapy was started with CBDCA area under the curve of 5 mg/mL·min on day 1 plus nab-PTX 70 mg/m^2^ on days 1, 8, and 15 once every 3 weeks under hospitalization. The treatment schedule, computed tomography images, and serum sodium levels are shown in Fig. 1. The serum sodium level immediately before the start of chemotherapy was 141 mmol/L. On day 8, hyponatremia was noted (serum sodium level: 119 mmol/L); however, symptoms of hyponatremia, such as fatigue, nausea, delirium, confusion, and seizures, were absent. The physician started oral sodium chloride 3 g/day (51 mEq/day of sodium) administration. The pharmacist confirmed that patient did not have decrease in body weight, increase in heart rate (82 beats per min), decrease of blood pressure (145/82 mmHg), and increase of hematocrit (39.5%). The patient’s blood urea nitrogen/creatinine ratio was 31.9 and serum albumin level immediately before SIADH onset was 3.6 g/dL. Based on this information, the pharmacist suspected chemotherapy-induced SIADH because of a lack of findings associated with changes in extracellular fluid volume and suggested the examination of plasma and urine osmolality and urinary sodium levels to the physician. The examination revealed the following findings: creatinine level, 1.06 mg/dL; plasma osmolality, 253 mOsm/kg; urine osmolality, 355 mOsm/kg; and urinary sodium level, 59 mEq/L. Based on these findings, a diagnosis of chemotherapy-induced SIADH was made. Serum cortisol and plasma vasopressin levels were not examined. The physician and pharmacist conferred and decided to continue chemotherapy with frequent monitoring of serum sodium levels. On day 15 of cycle 1, nab-PTX was suspended because of neutropenia. Subsequently, serum sodium levels improved to 139 mmol/L without additional treatment such as water restriction and continuous infusion, and oral administration of sodium chloride was discontinued on day 22. From cycle 2 onward, nab-PTX was reduced to 60 mg/m^2^ but was discontinued on day 59 due to neutropenia. Cycle 3 onward, CBDCA was also reduced to area under the curve 4.5 mg/mL·min, and treatment was suspended on days 94, 126, 155, and 162 owing to neutropenia. While repeating the partially suspended CBDCA or nab-PTX treatment due to neutropenia, five cycles of chemotherapy were completed. Regular computed tomography revealed a partial response throughout chemotherapy (Fig. 1). Furthermore, sodium levels did not decrease again throughout chemotherapy. The Naranjo Adverse Drug Reaction Probability Scale score was 5 points, categorized as “probable” (Table 2) [15].

**Table 2:** Naranjo adverse drug reaction probability scale

Factors	Yes	No	Do not know	Score
1. Are there previous conclusive reports on this reaction?	**+1**	0	0	+ 1
2. Did the adverse event appear after the suspected drug was administered?	**+2**	-1	0	+ 2
3. Did the adverse reaction improve when the drug was discontinued or a specific antagonist was administered?	+1	0	**0**	0
4. Did the adverse reaction reappear when the drug was readministered?	+2	**-1**	0	-1
5. Are there alternative causes (other than the drug) that could on their own have caused the reaction?	-1	** +2**	0	+ 2
6. Did the reaction reappear when a placebo was given?	-1	+ 1	**0**	0
7. Was the drug detected in the blood (or other fluids) in concentrations known to be toxic?	+ 1	0	**0**	0
8. Was the reaction more severe when the dose was increased, or less severe when the dose was decreased?	+ 1	0	**0**	0
9. Did the patient have a similar reaction to the same or similar drugs in any previous exposure?	+ 1	**0**	0	0
10. Was the adverse event confirmed by any objective evidence?	**+1**	0	0	+ 1
Total				5

9 points > : definite, 5–8 points: probable, 2–4 points: possible, 2 points < : doubtful

## Discussion and Conclusions

Here, we described a case in which the patient developed chemotherapy-induced SIADH but could continue CBDCA plus nab-PTX therapy without regimen changes because of early pharmacist intervention. Age of ≥ 60 years, female sex, body mass index of < 18.5 kg/m^2^, and baseline sodium level of < 135 mmol/L are risk factors for SIADH [16]. However, the only risk factor in our patient was age of ≥ 60 years. The diagnosis of SIADH may be difficult in patients using multiple concomitant drugs and with co-morbidities [13]. In fact, etiology was reported to be multifactorial in 51% of elderly patients with SIADH [17]. In addition, the mortality rate in hospitalized elderly patients with hyponatremia was 19% [17]. Given this evidence, physicians and pharmacists should consider the causes of SIADH from different perspectives, and the early detection of SIADH is important.

The most common malignancy associated with SIADH is small-cell lung cancer, accounting for 70% of all cancer-related SIADH cases [18]. Actually, SIADH is observed in 10–15% of all patients with small-cell lung cancer but only in 2–4% of all patients with non–small-cell lung cancer [19]. Cases of SIADH induced by allopurinol, famotidine, edoxaban, mefruside, limaprost alfadex, or triazolam have not yet been reported [20, 21]. Thiazides are common causative drugs for SIADH [22]. In the case of our patient, mefruside was administered for a long time and there were no physiological changes that would reach the plasma toxicity range of mefruside, suggesting that SIADH may not have been caused by mefruside. CBDCA and nab-PTX have been reported to be the causative drugs for SIADH [11, 23]. Several cases of chemotherapy-induced SIADH have been reported to commonly occur between 4 and 8 days after the first chemotherapy cycle [11, 12, 24]. Considering the evidence and the clinical course, chemotherapy may have been involved in the development of SIADH in this case. In addition, this case was classified as “probable” by the Naranjo Adverse Drug Reaction Probability Scale, indicating that CBDCA or nab-PTX was the causative drug for SIADH (Table 2).

The only definitive treatment for SIADH is elimination of its underlying cause [25]. Since SIADH recurrence after a second course of chemotherapy has also been reported [26], the regimen was changed after the onset of chemotherapy-induced SIADH in several cases [11, 12], and a regimen change might have been necessary in the case of our patient as well. However, because the patient tested negative for *EGFR* 19del, *EGFR* L858R, *BRAF* V600E, *KRAS* G12C, *ALK* fusion, *ROS1* fusion, *MET* exon 14 skipping, and *RET* fusion and the expression of programmed cell death protein ligand 1 was < 1%, only chemotherapy was available for this patient. Docetaxel, the recommended second-line treatment for non–small-cell lung cancer, was considered a candidate for the next regimen [27]. Overall survival has been reported to be longer with CBDCA plus nab-PTX than with docetaxel in patients aged 70 years and older with advanced squamous non–small-cell lung cancer [28]. In addition, the tumor size gradually decreased during course 2, as observed in computed tomography images obtained after treatment with the CBDCA plus nab-PTX regimen (Fig. 1). Thus, the present case report shows that in elderly patients with asymptomatic SIADH, it may be possible to continue CBDCA plus nab-PTX treatment with careful monitoring of sodium levels.

The CBDCA plus nab-PTX regimen can be administered as an outpatient treatment. Although pharmacists have an important role to play in the detection of adverse drug reactions and avoidance of their exacerbation [14, 29], SIADH cases with early intervention are limited. In recent years, the usefulness of pharmaceutical outpatient clinic in terms of chemotherapy has been demonstrated. Outpatient pharmaceutical interventions contribute to early detection of adverse drug reactions and avoidance of serious conditions [30, 31]. Collaboration between physicians and pharmacists in outpatient clinics may contribute to the early detection of chemotherapy-induced SIADH and continuation of chemotherapy.

This work had several limitations. First, the examination of hormones such as, plasma vasopressin, and cortisol was not performed during treatment. However, we ensured that there were no adrenal gland diseases in the patient’s medical history. Second, not all medical histories and drugs that could cause SIADH were excluded. Third, to the best of our knowledge, the specific mechanisms underlying SIADH induced by CBDCA and nab-PTX remain unclear, and no susceptibility factors have been identified. Therefore, it is uncertain whether the dose reduction of nab-PTX and CBDCA contributed to the prevention of SIADH recurrence. Finally, although the Naranjo Adverse Drug Reaction Probability Scale indicates a “probable” classification, the absence of SIADH recurrence with tumor shrinkage suggests that SIADH due to squamous cell carcinoma cannot be ruled out.

In summary, we encountered a case in which the patient developed chemotherapy-induced SIADH but was able to continue chemotherapy with early pharmacist intervention. This case has high clinical value in Japan, where elderly patients are receiving chemotherapy. In elderly patients without genetic mutations and few treatment options, even if they develop SIADH, chemotherapy should be continued with monitoring of serum sodium levels by physicians and pharmacists.

## Data Availability

No datasets were generated or analysed during the current study.

## Abbreviations

SIADH: Syndrome of inappropriate antidiuretic hormone secretion
CBDCA: Carboplatin
nab-PTX: Nab-paclitaxel
